# Novel paediatric case of a spinal high-grade astrocytoma with piloid features in a patient with Noonan Syndrome

**DOI:** 10.1038/s41698-024-00734-3

**Published:** 2024-10-19

**Authors:** Jordan Staunton, Pamela Ajuyah, Angela Harris, Chelsea Mayoh, Marie Wong, Megan Rumford, Patricia J. Sullivan, Paul G. Ekert, Noemi Fuentes-Bolanos, Mark J. Cowley, Loretta M. S. Lau, David S. Ziegler, Paulette Barahona, Neevika Manoharan

**Affiliations:** 1 Kids Cancer Centre, Sydney Children’s Hospital, Randwick, NSW Australia; 2 Children’s Cancer Institute, Lowy Cancer Centre, UNSW Sydney, Kensington, NSW Australia; 3 Department of Anatomical Pathology, NSW Health Pathology, Prince of Wales Hospital, Randwick, NSW Australia; 4 School of Clinical Medicine, UNSW Medicine & Health, UNSW Sydney, Sydney, NSW Australia; 5 University of New South Wales Centre for Childhood Cancer Research, UNSW Sydney, Sydney, NSW Australia; 6 Murdoch Children’s Research Institute, Royal Children’s Hospital, Parkville, VIC Australia; 7 Cancer Immunology Program, Peter MacCallum Cancer Centre, Melbourne, VIC Australia; 8 Sir Peter MacCallum Department of Oncology, The University of Melbourne, Parkville, VIC Australia

**Keywords:** CNS cancer, Cancer genomics

## Abstract

Noonan Syndrome (NS) is associated with an increased risk of low-grade central nervous system tumours in children but only very rarely associated with high-grade gliomas. Here we describe the first reported case of a spinal high-grade astrocytoma with piloid features (HGAP) in a child with NS. This case was a diagnostic and treatment dilemma, prior to whole-genome germline and tumour sequencing, tumour transcriptome sequencing and DNA methylation analysis. The methylation profile matched strongly with HGAP and sequencing identified somatic *FGFR1* and *NF1* variants and a *PTPN11* germline pathogenic variant. Therapeutic targets were identified but also alterations novel to HGAP such as differential expression of *VEGFA* and *PD-L1*. The germline *PTPN11* finding has not been previously described in individuals with HGAP. This case underscores the power of precision medicine from a diagnostic, therapeutic and clinical management perspective, and describes an association between HGAP and NS which has not previously been reported.

## Introduction

Noonan Syndrome (NS) is an autosomal dominant genetic disorder which predisposes to the development of cancer^1^. Clinically, the disorder is characterised by distinctive facial features, short stature, congenital heart disease, lymphatic malformations, renal anomalies, bleeding diatheses and developmental delay^1^. The genes implicated in NS encode proteins which are integral to the RAS-MAPK pathway, which controls cell proliferation, survival and differentiation^1^ and the risk of malignancy in NS is approximately 4% by the age of 20 years^2^. The vast majority of *PTPN11*-associated central nervous system (CNS) tumours described in the literature are low-grade glial or glioneuronal tumours^2^. There are only three previously reported cases of high-grade gliomas in patients with NS, only one of which was a spinal cord tumour^3,4^.

Here, we report a patient with NS and a spinal cord tumour, whose diagnosis could not be made by conventional histopathological methods. His tumour underwent whole genome sequencing (WGS) (paired with germline analysis), tumour transcriptome sequencing and DNA methylation analysis to reach a diagnosis of a high-grade astrocytoma with piloid features (HGAP). This represents the first reported case of HGAP described in a patient with NS.

HGAP is a new CNS tumour diagnostic category, as defined in the 5th edition of the WHO classification of CNS tumours. Tumours which fit in this category were primarily previously defined as anaplastic astrocytomas with piloid features (and earlier as anaplastic pilocytic astrocytomas). Reinhardt et al. performed DNA methylation profiling of 102 histologically defined anaplastic pilocytic astrocytomas to find that 83 tumours shared a common methylation profile which was distinct from the glioma reference classes^5^. HGAP has a wide range of described histological features, and as such requires this methylation profile for definitive diagnosis of this tumour type^6^.

## Results

### Clinical background

The patient was a 17 year old boy, who presented with a one month history of reduced mobility. He had a known de novo clinical and molecular diagnosis of *PTPN11*-associated NS (c.218 C > T) and multiple co-morbidities. These included cerebral palsy (GMFCS 4, MACS 3), intellectual disability, and hearing and visual impairment^7,8^. He was born at 35 weeks gestation and suffered a neonatal intraventricular haemorrhage, leading to subsequent hydrocephalus, shunted via a ventriculo-peritoneal shunt. As an infant he had refractory immune thrombocytopenia and underwent a splenectomy. He was gastrostomy fed and had a personal history of other resolved co-morbidities including gastroesophageal reflux requiring fundoplication, epilepsy and undescended testes.

**Fig. 1:**
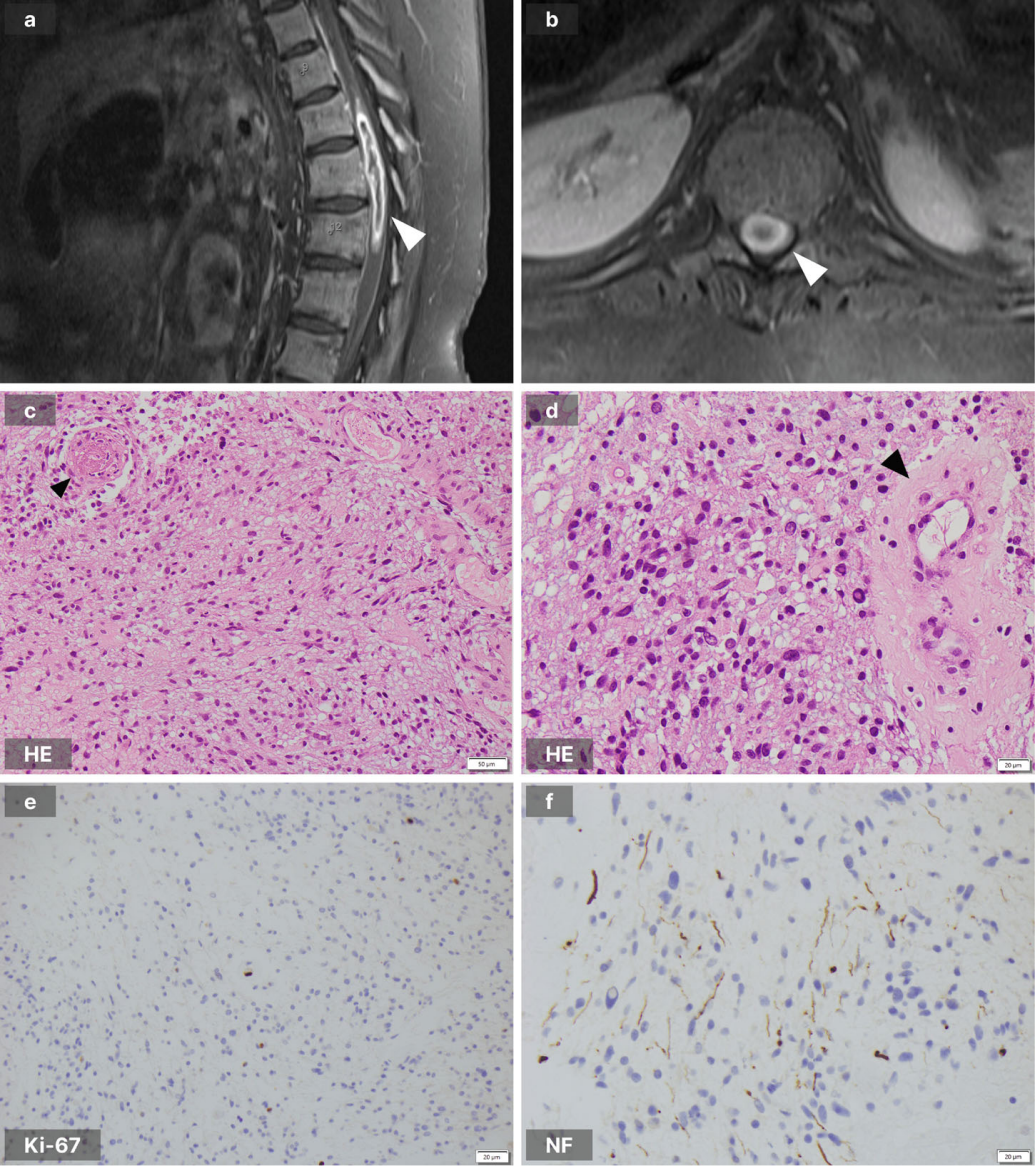
MRI brain and whole spine and pathology images. **a** T1-weighted sagittal sequence of the thoraco-lumbar spine demonstrating the peripherally enhancing lesion (white arrowhead). **b** T1-weighted transverse sequence at the level of the lower thoracic spine showing the lesion to expand the spinal cord (white arrowhead). **c** Haematoxylin and eosin (HE) stain demonstrating tumour cells with mild cytological atypia and a small blood vessel containing a fibrin thrombus (black arrowhead). **d** HE stain showing tumour cells and a hyalinised blood vessel (black arrowhead). **e** Ki-67 stain demonstrating rare positive tumour cells ( < 1%). **f** Neurofilament (NF68) stain demonstrating the infiltrative nature of the tumour.

Over the month prior to presentation, the patient’s mobility deteriorated from independent crawling and wheelchair transfers to complete lower limb paralysis. He was unsettled, lethargic and was experiencing severe constipation. On clinical examination, he was persistently tachycardic and had complete paralysis of his lower limbs. There were no features of neurofibromatosis type 1 on examination, such as café au lait macules, axillary or inguinal freckling, neurofibromas or distinctive osseous lesions. He underwent an MRI brain and whole spine which identified an expansile, peripherally enhancing lesion in the lower spinal cord extending from T10-T12/L1, with resultant cord oedema, extending superiorly to C6/C7 and inferiorly to the tip of the conus (Fig. 1a, b). These appearances were concerning for a high-grade spinal cord glioma.

The patient proceeded to a T10-T12 laminectomy and resection of the intramedullary spinal cord lesion. Macroscopic intra-operative appearance had features indicating the potential for a high-grade lesion. Intraoperative frozen section assessment demonstrated a mildly pleomorphic glial tumour with no high-grade features. Post-operative imaging revealed residual regions of enhancement at the level of the T10/T11 disc and at the level of upper T12, suggesting residual tumour in these regions.

### Histopathology

Histopathological features were of a moderately hypercellular astrocytoma (GFAP, Olig2 immunohistochemical staining positive) with mild cytological atypia and focal infiltrative growth (demonstrated with neurofilament NF68 staining, Fig. 1f). Mitoses were rare and the Ki-67 proliferative index was low (visually estimated at less than 1%). There were no piloid features, no Rosenthal fibres, and no eosinophilic granular bodies. There was focal, non-palisading necrosis, scattered hyalinised vessels, and microvascular proliferation involving rare vessels only. Several vessels showed fibrin thrombi with associated perivascular haemorrhage and inflammation (Fig. 1c–f). In consideration with the finding of only mild atypia and rare mitoses, the scant necrosis and rare foci of microvascular proliferation were interpreted as changes secondary to vascular thrombosis and infarction. The overall morphological features were considered best in keeping with a low-grade glioma (likely CNS WHO grade 2), but requiring molecular studies for a definitive integrated diagnosis. The clinical history, radiological features and gross surgical features however raised concern about an unrecognised high-grade nature to this tumour. Increasingly it is now recognised, as demonstrated by Sturm et al., that prospective multi-omic integration assists in the diagnosis and management of paediatric brain tumour patients^9^.

### Genomic profile

For diagnostic clarity the stored tumour sample, paired with a peripheral blood sample (germline biospecimen), were submitted for comprehensive genomic profiling through the Australian PRecISion Medicine for Children with Cancer trial (PRISM, NCT03336931). Table 1 summarises the genomic findings.

**Table 1:** Molecular features of zccs1703

Tumour Purity (%)		74
Tumour mutational burden (SNVs/exome)		11
Single nucleotide variants	Somatic DNA	NM_023110.3(FGFR1):c.1638 C > A (p.Asn546Lys) NM_000267.3(NF1):c.655-6 T > G. NM_000267.3(NF1):c.4604_4619del (p.Asp1535AlafsTer13)
	Germline DNA	NM_002834.4(PTPN11):c.218 C > T (p.Thr73Ile)
Copy number variants		Biallelic deletion of 9p21.1-21.3 - includes CDKN2A/B (0 copies) CD274 gain (4 copies)
Expression (TPM)	CDKN2A	4.01 (*p* = 0.251) Cohort Mean: 41.66
	CDKN2B	2.69 (*p* = 0.269) Cohort Mean: 21.15
	VEGFA	997.44 (*p* = 0.041) Cohort Mean: 213.07
	CD274/PD-L1	34.84 (*p* = 0.002) Cohort Mean: 4.03
DKFZ Methylation Classifier Findings (v11b.4)		Anaplastic pilocytic astrocytoma (0.88)
DKFZ Methylation Classifier Findings (v12.5)		High-grade astrocytoma with piloid features (0.99)
MGMT promoter methylation status		Unmethylated

zccs1703 represents the index patient of this case report.

DNA methylation analysis (performed using the MNP brain classifier version 12.5 https://www.molecularneuropathology.org/mnp/classifiers/11) was a strong match for HGAP (score 0.99) (Table 1)^10^. This methylation match satisfied the WHO Classification of Central Nervous System Tumours (5th ed.) diagnostic criteria for HGAP, which requires an astrocytic glioma with a DNA methylation profile HGAP. Desirable criteria are MAPK pathway gene alterations and homozygous deletion or mutation of *CDKN2A* and/or *CDKN2B* (both present in this tumour), as well as mutation of *ATRX* and anaplastic histological features (both not present in this tumour). DNA methylation profiling currently defines this subgroup of CNS tumours and is the only method to definitively establish this diagnosis^5^. The integrated diagnosis of HGAP was therefore made on the basis of a methylation match and results were returned to the treating medical oncologist within 8 weeks of his operation. An important differential diagnosis to exclude was a diffuse midline glioma, H3 K27-altered. This tumour had retained H3 p.K27me3 (K27me3) on immunohistochemistry (the loss of which is an essential diagnostic criterion for this entity) as well as a negative nuclear staining for the H3K27M protein.

**Fig. 2:**
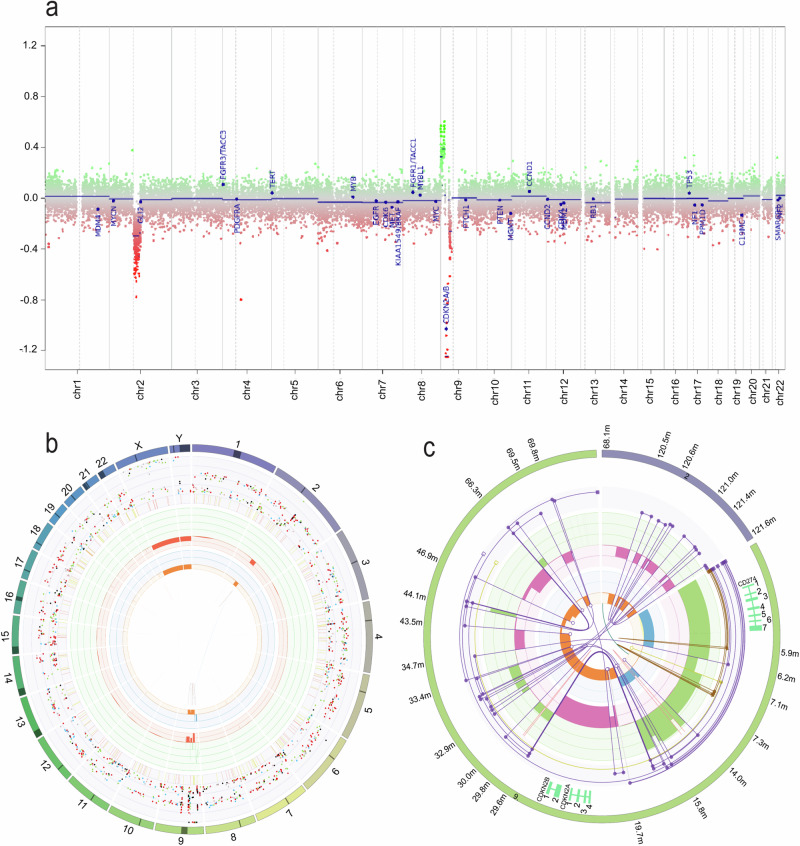
Genomic profile of zccs1703. **a** Copy-number profile from 850 K methylation array data, generated by the MNP DNA methylation classifier. **b** CIRCOS plot demonstrating somatic single nucleotide variants (SNVs), indels, chromosomal changes and structural rearrangements from WGS data, generated by PURPLE^29^. This outer ring shows the 24 chromosomes, followed by SNVs represented by coloured dots in the sequential inner ring (C > A blue, C > G black, C > T red, T > A grey, T > A grey, T > C green, T > G pink), with the y-axis representing the purity-adjusted variant allele frequency. The next ring represents short insertions (yellow) and deletions (red). The green inner ring displays copy number gains and the red inner ring copy number losses. The rings with orange and blue shading demonstrate the minor allele copy number, where loss of heterozygosity (i.e., minor allele copy number of 0) is indicated in orange, whereas amplification of both alleles is shown in blue. The innermost circle represents different types of structural variants in the tumour (translocations (blue), deletions (red), insertions (yellow), tandem duplications (green) and inversions (black)). **c** LINX plot highlighting the structure of a complex chain of structural variants from chromosomes 2 and 9, revealing biallelic deletion of *CDKN2A* and *CDKN2B*, and copy number gains of *CD274*. The LINX algorithm takes the copy number of distinct genomic segments (green and magenta), each segment’s minor allele ploidy (orange and blue) and structural variants (purple), then annotates segments with genes of interest (outer track), clusters the events based on genomic proximity and variant allele frequency and chains the integrated segments, to reveal predicted derivative chromosomes (purple chains). A detailed guide to interpret these plots is in the LINX publication^15^.

**Fig. 3:**
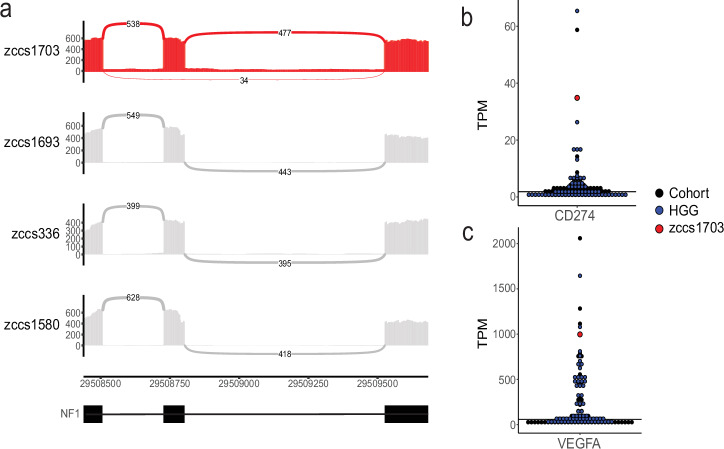
RNA sequencing findings of zccs1703. **a** Sashimi plot demonstrating altered splicing by the *NF1* splice region variant (c.655-6 T > G). From top to bottom, tumour RNA of zccs1703, zccs1693 (pilocytic astrocytoma), zccs336 (pilocytic astrocytoma), zccs1580 (HGG H3 & IDH wildtype). **b** Relative expression of CD274 (*PD-L1*) (TPM) in this patient (zccs1703: red) compared to other HGG (high-grade glioma: blue) and all gliomas (cohort: black). **c** Relative expression of *VEGFA* (TPM) in this patient (zccs1703: red) compared to other HGG (blue) and all gliomas (cohort: black). TPM: transcript per million, HGG: high-grade glioma.

Additional supportive molecular features of HGAP in the tumour included multiple structural complexities within chromosomes 2 and 9 (Fig. 2) and numerous hits to the MAPK signalling pathway. A pathogenic somatic gain-of-function *FGFR1* p.N546K variant was found and is well described in the literature including in the context of HGAP^5^. The tumour also contained two loss-of-function variants in *NF1* which were a deletion variant (c.4604_4619del (p.Asp1535AlafsTer13)) in exon 34/57 leading to a premature stop codon and a splice region variant (c.655-6 T > G) in intron 6, predicted to be splice altering by *Introme*^11^, and observed to cause exon skipping in 6% (24/398) of RNA-sequencing reads (Fig. 3a). Germline and somatic *NF1* variants have been reported in HGAP^5,12^. Cimino et al. reported that 9% of an expanded cohort of HGAP patients have a diagnosis of NF1 syndrome, including two children^12^. In this case manual curation of germline data confirmed that the origin of the two *NF1* variants was indeed somatic (Supplemental Fig. 1). As *NF1* is a negative regulator of the MAPK pathway, this complete loss of *NF1* would result in MAPK pathway activation. This is therefore also in agreeance with the methylation class of HGAP, which is a subclass within the superfamily ‘diffuse glioma, MAPK altered, cell-cycle activated’. No differential RNA expression was identified in the genes downstream in the MAPK signalling pathway, which is not atypical in this context.

This patient’s tumour had a 9.7 Mb biallelic deletion within the 9p21.1-21.3 region which contains the *CDKN2A* and *CDKN2B* loci and was associated with low RNA expression of these genes (Fig. 2, Table 1). CDKN2A and CDKN2B regulate the cell cycle and are deleted across a wide variety of paediatric tumours^2^ including 80% of HGAP^5^. RNA expression analysis of the tumour showed aberrant expression of *VEGFA*, a growth factor actively involved in vascular development^13^ (997.44 TPM, *p* = 0.041) and *CD274* (also known as *PD-L1*), an immune checkpoint inhibitor^14^ (34.84 TPM, *p* = 0.002) (Fig. 3b, c). Both the *CDKN2A/B* deletion and *CD274* amplification appear to have been generated on the same derivative chromosome, from a complex cluster of structural variants (Fig. 2c), as revealed by LINX analysis^15^. *PD-L1* immunohistochemistry (clone SP263) was positive, with 80-90% of the tumour cells showing diffuse/fibrillary cytoplasmic staining, consistent with the RNA-seq findings. Germline WGS analysis confirmed the previously identified heterozygous pathogenic variant in *PTPN11*. *PTPN11* encodes a protein tyrosine phosphatase that regulates the MAPK signalling pathway^16,17^. This missense variant (T73I) is in a mutational hotspot and has been associated to NS phenotype in numerous cases in the literature^18–24^.

### Treatment and follow-up

Since diagnosis, the patient has continued to experience multiple complications. He has had persistent loss of function of his lower limbs and had a prolonged admission to ICU with respiratory failure and sepsis. Therapeutic options are limited for this patient given his comorbidities, his profound neurological disability and the impact of medical procedures on his quality of life. To minimise hospitalisation and maximise this quality of life, his guardian and treating team chose to delay therapeutic intervention until progression or relapse. Subsequently, this patient experienced stable disease for five months after his operation prior to disease progression. He was treated with intensity-modulated radiation therapy, 4560 cGy in 19 fractions. He has remained clinically stable subsequently and is having surveillance MRI scans as follow-up.

## Discussion

Here we describe a novel report of HGAP in a patient with NS which serves to highlight the role of integrative genomic profiling in aiding diagnostics and therapeutic considerations in paediatric neuro-oncology.

**Fig. 4:**
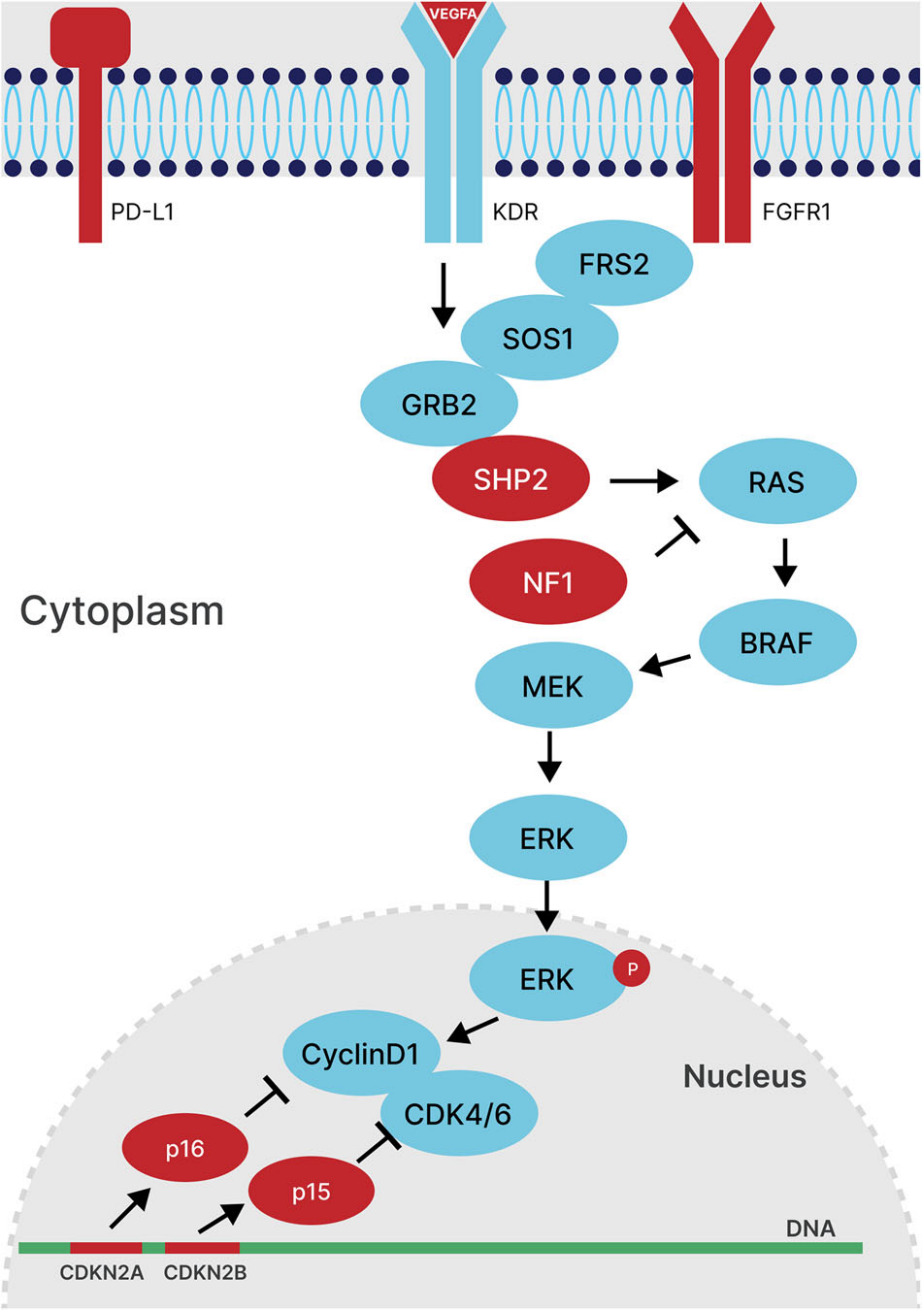
MAPK pathway activation in zccs1703. Pathway summary of altered genes in this case with a focus on the MAPK signalling pathway. Mutated genes in this patient are coloured red and unaffected genes are coloured blue.

Genomic analysis of HGAP tumours has demonstrated a propensity for somatic MAPK pathway activation with *NF1* variants being the most common followed by *FGFR1*^5,12^. The vast majority of HGAP tumours appear to have a singular MAPK pathway aberration, with reports of only 7% (8/120) of HGAP with two or more alterations^5,12^. This case therefore adds to that minority, with four aberrations involving the MAPK pathway (somatic *FGFR1* and *NF1* x2, germline *PTPN11*) (Fig. 4).

It has been proposed that the molecular profile of paediatric HGAP may differ from adult HGAP. Within a subgroup of seven paediatric HGAP there was only one case with *CDKN2A* deletion, and all cases had retained *ATRX*^25^. In contrast, within adult HGAP, approximately half of the cases reported are shown to have *ATRX* loss^5,12^. Our case did not demonstrate *ATRX* loss and adds to the relatively small number of paediatric HGAP in the literature^12,25^. This case report therefore provides additional evidence to assist in defining the supportive molecular features of paediatric HGAP.

In isolation, these somatic molecular alterations identified in the tumour of this patient would not be sufficient to diagnose this tumour as a HGAP due to the promiscuity of these genes in high- and low-grade CNS tumours. However, in combination with the definitive methylation match, these findings were declarative in this diagnostic dilemma and were fundamental in dictating his clinical management. Through the implementation of a comprehensive sequencing platform, our study has uncovered the previously unreported gene targets *VEGFA* and *PD-L1*. This finding of elevated expression adds valuable insights to the existing knowledge base of transcriptomics in HGAP tumours. This RNA overexpression could also indicate potential activity of anti-VEGFA antibodies and immune checkpoint inhibitors, both of which provide further options for personalised targeted therapy.

This case report is the first documented case of HGAP in NS, thereby expanding our knowledge of the syndromes that may be associated with this rare subgroup. There is a documented association between neurofibromatosis type 1^5,12^ and HGAP. CNS tumours are described in patients with NS, though remaining relatively rare. Low-grade gliomas predominate, with only three previously described cases of high-grade gliomas in patients with NS^2,4,3^. Given the recent incorporation of the HGAP diagnosis into the 5th edition (2021) of the WHO classification of CNS tumours^6^ and the minimal requirement for diagnosis being a positive methylation match, we postulate that there may be more paediatric patients with NS and CNS tumours whose tumours could be more accurately re-classified as HGAP.

NS infers an approximate cumulative cancer risk of 23% up to age 55 years^26^. This represents a 3.5-fold increased risk compared to the general population, with haematological malignancies being the most common cancer in this cohort^26^. Despite these known tumour and malignancy associations, there is no established surveillance program for persons with NS. Due to the heterogeneous phenotype and complex underlying molecular cause, it is challenging to confirm that surveillance in this population will be beneficial. Based on this case and the literature on CNS tumours in patients with NS it would be pertinent for clinicians of patients with NS to carefully consider neurological symptoms that may herald the presence of a CNS tumour and ensure accurate histologic and molecular diagnosis of any identified lesions (including HGAP in the differential diagnosis).

The utility of comprehensive genomic profiling in this case was not only to assist with accurate clinical diagnosis but also to help inform potential therapeutic avenues. As is common in children with NS, our patient has complex medical and social co-morbidities and all therapeutic possibilities needed to be carefully considered as to what would be the most appropriate. This crucial change in diagnosis from a likely low-grade glioma to high-grade glioma was paradigm-changing for this patient’s treatment strategy, and unfortunately confers a far graver prognosis^27^. This knowledge enabled us to help balance his quality of life appropriately with any expected treatment outcome. Through the PRISM trial and Molecular Tumour Board, other targeted therapeutic considerations were identified. These included a MEK inhibitor for the *FGFR1* and *NF1* alterations, combination MEK inhibitor with a CDK4/6 inhibitor (considering the bi-allelic loss of *CDKN2A* and *CDKN2B*), immune checkpoint inhibitor for high *PD-L1* expression and bevacizumab (anti-VEGFA antibody) for high *VEGFA* expression. At present the patient is on imaging surveillance after completing intensity-modulated radiation therapy at first progression, but now has multiple therapeutic considerations available in the setting of future relapsed or refractory disease.

In summary, this case adds to the small body of literature regarding high-grade gliomas in NS as well as paediatric HGAP and is the first reported case of a HGAP in a patient with NS. This report highlights the utility of comprehensive genomic profiling in order to aid accurate clinical diagnosis and treatment, which was integral to the appropriate management of this child.

## Methods

### Study design

The PRISM trial (ClinicalTrials.gov registration: NCT03336931) was a multicenter prospective observational cohort study conducted by the Australian ZERO Childhood Cancer Precision Medicine Program and was open from September 2017. Patients were recruited between September 2017 and December 2020. All clinical data was collected by designated clinical research associates and clinicians based at each of the eight paediatric oncology centres in Australia.

### Study oversight

The study was conducted in accordance with Good Clinical Practice guidelines and the Declaration of Helsinki and approved by the Hunter New England Human Research Ethics Committee of the Hunter New England Local Health District in Australia (reference no. 2019/ETH00701). Written informed consent for this patient was provided by their legal guardian. There was no participant compensation. Informed consent for this publication was also provided by the legal guardian of the participant.

### Patient and tumour samples

This patient was enrolled on the study under Cohort B (The PRISM Rare Cancer Cohort). Patients younger than 21 years with either a rare cancer of uncertain prognosis and no established treatment strategy, or a tumour where histopathological examination has not been able to establish a diagnosis could be consented and registered on the study. After trial registration, this patient’s samples were delivered to the central laboratory at the Children’s Cancer Institute (CCI) (Sydney) for processing. Tumour tissue was fresh, snap-frozen or cryopreserved on receipt. Clinical and demographic data at registration and follow-up were entered into the Labmatrix by Biofortis v.R7 3.2.0 laboratory information management system.

### Molecular profiling

Whole genome sequencing (paired tumour-germline), whole transcriptome sequencing (WTS) and DNA methylation analysis was performed. The analytical pipelines for molecular profiling and variant curation for WGS, WTS and methylation, have been described previously^28^.

## Supplementary information

Supplementary Information File

## Data Availability

The WGS, RNA-seq and DNA methylation data generated by this study are available from the European Genome-phenome Archive under accession no. EGAS00001007937. This data is available by request through the ZERO Data Access Committee (ZERO DAC) and approved by the ZERO Data Access Committee by emailing zeroDAC@ccia.org.au.

## Code availability

The code used for the molecular analysis has been previously reported^28^ and is available at https://github.com/CCICB/2020-hrPC-landscape.
