# Supplementary material for: Novel paediatric case of a spinal high-grade astrocytoma with piloid features in a patient with Noonan Syndrome
Source: NPJ Precis Oncol. 2024 Oct 19;8:236. doi: 10.1038/s41698-024-00734-3 (PMC11490609; doi:10.1038/s41698-024-00734-3)
Supplement: Supplementary file 1 — Supplementary Information File [file 41698_2024_734_MOESM1_ESM.pdf]

SUPPLEMENTARY INFORMATION FILE

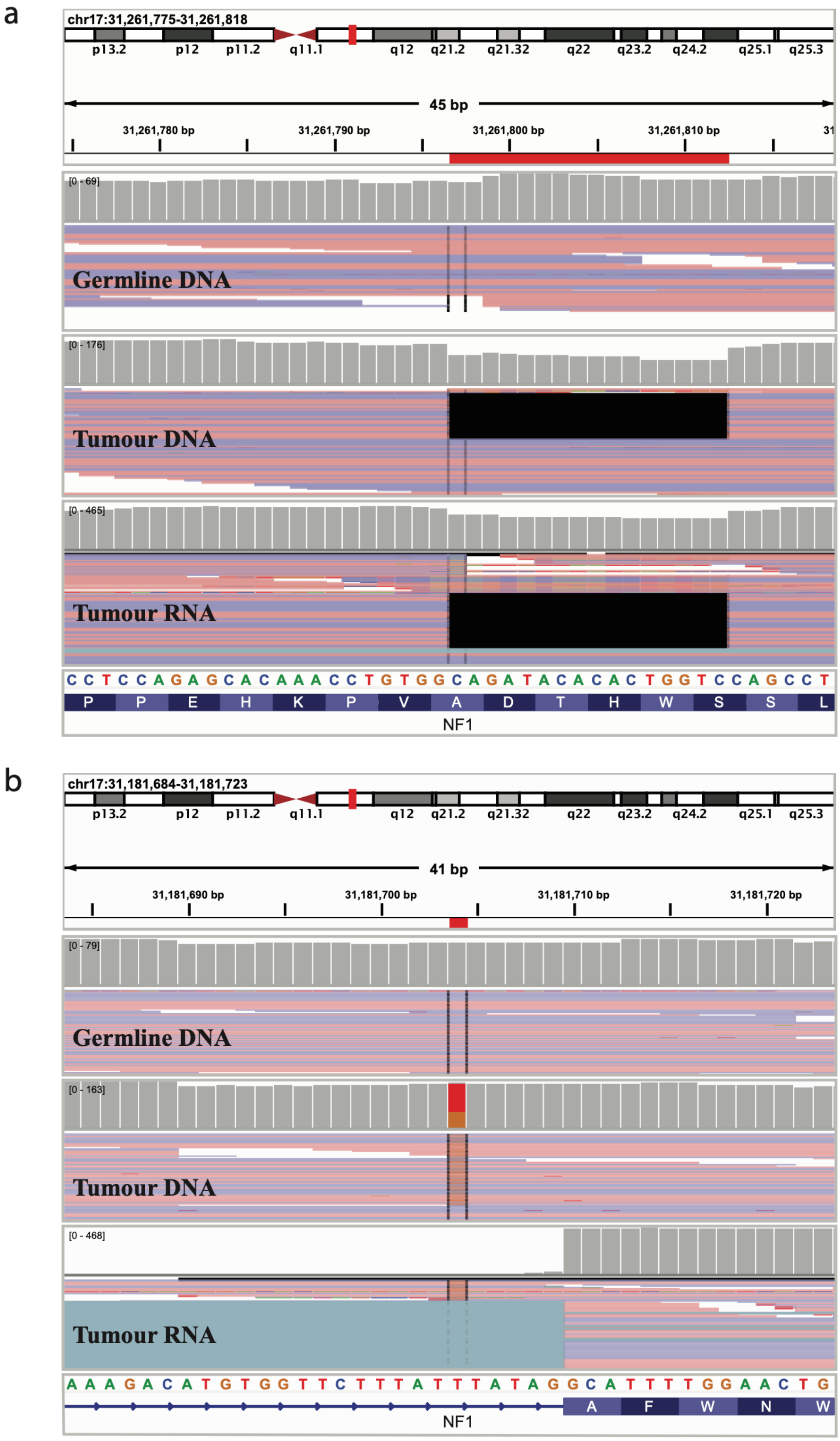

Supplemental Figure 1. IGV images of the NF1 variants in zccs1703.

The grey segment is the sequencing coverage, the pink lines represent forward reads, the purple lines reverse reads and the teal lines in the tumour RNA track represent regions not in the mature RNA transcript (introns, deletions). The coloured bases within the tracks show misaligned bases to the reference genome. **a)** NF1 splice region variant (c.655-6T>G) - the top track is the germline DNA, the middle track is the tumour DNA and the bottom track is the tumour RNA. Reads in RNA containing T>G mutation arise from pre-mRNA **b)** NF1 deletion variant (c.4604_4619del (p.Asp1535AlafsTer13)) - the top track is the germline DNA, the middle track is the tumour DNA and the bottom track is the tumour RNA. Coloured bases are soft clipped which occurs when the deletion variant is at the end of the read, and thus add further support to the deletion variant.
